# Neural Network Assisted Inverse Dynamic Guidance for Terminally Constrained Entry Flight

**DOI:** 10.1155/2014/686040

**Published:** 2014-02-26

**Authors:** Hao Zhou, Tawfiqur Rahman, Wanchun Chen

**Affiliations:** School of Astronautics, Beihang University, Beijing 100191, China

## Abstract

This paper presents a neural network assisted entry guidance law that is designed by applying Bézier approximation. It is shown that a fully constrained approximation of a reference trajectory can be made by using the Bézier curve. Applying this approximation, an inverse dynamic system for an entry flight is solved to generate guidance command. The guidance solution thus gotten ensures terminal constraints for position, flight path, and azimuth angle. In order to ensure terminal velocity constraint, a prediction of the terminal velocity is required, based on which, the approximated Bézier curve is adjusted. An artificial neural network is used for this prediction of the terminal velocity. The method enables faster implementation in achieving fully constrained entry flight. Results from simulations indicate improved performance of the neural network assisted method. The scheme is expected to have prospect for further research on automated onboard control of terminal velocity for both reentry and terminal guidance laws.

## 1. Introduction

In the past half century, entry guidance law has been of particular interest for research. One of the reasons for this growing importance is the rise in missions to other planets, which is further emphasized by recent endeavor for prompt global strike capability. Although present day guidance technologies are able to attain very high precision in conventional guided munitions, the same cannot be said for planetary entry vehicles. This is partly because in entry guidance certain path constraints are considered, which are not considered for conventional munition guidance. These path constraints cannot be violated in entry flight; as such, their satisfaction is the primary concern in such guidance. Due to this unavoidable reason, some terminal constraints seem to have been compromised in many entry guidance laws. However, with the growing requirement for terminally more accurate entry mission, there is a need to address these terminal constraints with much more weightage. With this in mind, an entry guidance law is presented, which, under a wide range of uncertainty, appears to be able to satisfy terminal constraints in position, velocity, and angular states as well as comply with the hard path constraints.

Entry guidance law was first successfully applied in the Apollo program [1, 2], which was a reference tracking method. Thenceforth, an array of guidance techniques based on reference tracking has followed [1, 3–10]. Essentially, these methods are applications of different optimal control laws, such as linear quadratic regulator (LQR) [4, 5], state dependent Riccati equation (SDRE) [6], feedback linearization [7, 11], and pseudospectral control laws [9, 10]. These methods strictly follow an offline trajectory without consideration for onboard regulation of terminal states. As a result, it is preferred to have a guidance law that is capable of addressing unforeseen requirements for vehicle trajectory and it terminal states. From this necessity, an alternate category of guidance laws was developed. These methods predict the terminal state and correct the control command accordingly. Such methods are categorized as “predictor-corrector” guidance laws [12–16]. A number of entry guidance laws have been designed using similar principle and these differ from each other in their method of the prediction and subsequent derivation of guidance correction. Although this genre of guidance law eliminates the need for precomputed reference profile, it lacks robustness due to difficulty in enforcing path constraints. Moreover, the onboard prediction process is computationally engaging.

With future planetary entry and global payload delivery mission in consideration, neither precomputed profile tracking nor predictor-corrector guidance law seems capable enough in achieving a multiconstrained entry flight. Guidance laws that depend on a precomputed profile are assumed to be more susceptible to error in the presence of atmospheric and aerodynamic uncertainty. However, in such guidance laws path constraints can be easily satisfied. On the other hand, predictor-corrector guidance laws could be suggested to be more autonomous, yet involve highly demanding onboard computation. Under these circumstances, it is believed that a guidance law would be better suited for future requirements if it involved a simple computational method for onboard modification of an offline profile that complies with path constraints. This paper presents a guidance law that is designed to work in such manner. The proposed law uses a three-dimensional Bézier curve in approximating the vehicle trajectory. It is shown that this approximation significantly simplifies both offline reference generation and onboard trajectory correction. The properties of Bézier curve are employed to enforce initial and terminal states and path constraints. This constrained approximation is then used in generating the guidance command. The solution thus attained readily satisfies terminal position and angular constraints. However, for the satisfaction of terminal velocity constraint, the method requires to predict the terminal velocity under disturbance. At a previous work as reported in [17], the prediction was made using a full state simulation. In this paper, a neural network is utilized for the online prediction of the terminal velocity. This significantly reduces the total runtime of the guidance law. There is every hope that the entry guidance law presented here could not only aid in achieving higher accuracy in planetary landing missions but also prove to be significant in the development of global payload delivery vehicles.

## 2. Problem Statement

The focus of the paper is to present a guidance law for atmospheric entry flight. An atmospheric entry flight usually starts at an altitude of 120 km and terminates at around 25 to 30 km. However, from the literature it can be seen that the majority of the guidance laws are designed to actively guide the entry vehicle from 55 to 50 km down to 30 to 25 km, which is due to the ineffectiveness of control measure over the altitude of 60 km. Some of the guidance methods adopt a quasiequilibrium glide (QEG) flight under the assumption of the quasiequilibrium glide condition (QEGC) [13, 18, 19]. This condition defines a feasible flight corridor in the altitude-velocity space. Flight within this corridor has been mathematically found to ensure satisfaction of the associated path constraints. Adopting the same conditions, the guidance law is formulated to guide an entry vehicle from an altitude of 50 km and hand it over to terminal area energy management (TAEM) system at an altitude of 25 km.

### 2.1. Trajectory Dynamics

For the trajectory dynamics, a point mass vehicle model over a round earth is adopted. The rotating earth effects can be assumed to be compensated by the feedback nature of the guidance law. Thus, the three-degree-of-freedom (3DOF) dynamics of a point mass entry vehicle model (in a geodetic coordinate frame) can be described through the following equations of motion:

(1) $$\begin{array}{c} \dot{r}=v\sin\gamma , \end{array}$$

(2) $$\begin{array}{c} \dot{\theta }=\frac{\left(v\cos\gamma \sin\psi \right)}{\left(r\cos\varphi \right)}, \end{array}$$

(3) $$\begin{array}{c} \dot{\varphi }=\frac{v\cos\gamma \cos\psi }{r}, \end{array}$$

(4) $$\begin{array}{c} \dot{v}=\frac{D}{m}-g\sin\gamma , \end{array}$$

(5) $$\begin{array}{c} \dot{\gamma }=\frac{L\cos\sigma }{\left(mv\right)}-\left(\frac{g\cos\gamma }{v}\right)+\left(\frac{v\cos\gamma }{r}\right), \end{array}$$

(6) $$\begin{array}{c} \dot{\psi }=\frac{L\sin\sigma }{\left(mv\cos\gamma \right)}+\frac{v\cos\gamma \sin\psi \tan\varphi }{r}. \end{array}$$

In the above equations, the position of the vehicle is defined by the parameters *r*, *θ*, and *φ* which denote the radial distance, longitude, and latitude, respectively. Vehicle dynamics is represented by *v* (velocity in m/sec), *γ* (flight path in radians), and *ψ* (azimuth angle in radians). The other terms *m*, *g*, *L*, and *D* stand for vehicle mass (kg), gravitational acceleration, lift, and drag, respectively. The aerodynamic forces are defined as

(7) $$\begin{array}{c} L=0.5\rho v^{2}S_{\text{ref}}c_{l},\\ D=0.5\rho v^{2}S_{\text{ref}}c_{d}, \end{array}$$

where *ρ* is atmospheric density and *c*_*l*_ and *c*_*d*_ are the coefficients of lift and drag, respectively.

### 2.2. Quasiequilibrium Glide Condition

In the QEG flight, the vertical component of acceleration and the flight path angle are assumed to be small. Under this supposition, setting *γ* = 0 and *dγ*/*dt* = *ε* (very small) in (5) gives the following QEG*C* expression:

(8) $$\begin{array}{c} \frac{L\cos\sigma }{\left(mv\right)}-\left(\frac{g\cos\gamma }{v}\right)+\left(\frac{V\cos\gamma }{r}\right)=\varepsilon . \end{array}$$

The above expression of the QEGC is valid within a specific range of altitude and velocity. Smooth transition to this condition requires the satisfaction of another condition, which involves the slopes of the trajectories before and after the transition. Mathematically, it can be stated as in (9), where *dr*/*dv* is the slope of entry trajectory and *dr*/*dv*∣_QEGC_ is the slope of a QEG flight trajectory. Once (9) is satisfied for a very small positive value of *δ*_0_(*δ*_0_ > 0), the transition to QEG is expected to be smooth and feasible. Consider

(9) $$\begin{array}{c} \left|{\frac{dr}{dv}-\frac{dr}{dv}|}_{\text{QEGC}}\right|<\delta_{0}. \end{array}$$

The slope of a QEG trajectory (*dr*/*dv*∣_QEG*C*_) is expressed as follows where *H*_*s*_ is the scale altitude:

(10) $$\begin{array}{c} {\frac{dr}{dv}|}_{\text{QEGC}}=\frac{vH_{s}{\left(2m\cos\gamma +c_{l}\rho S_{\text{ref}}r\cos\sigma \right)}^{2}}{mg\cos\gamma \left(2mH_{s}\cos\gamma +c_{l}\rho S_{\text{ref}}r^{2}\cos\sigma \right)}. \end{array}$$

The entry from an altitude of 120 km begins with a fixed angle of attack and a zero bank angle (a higher bank angle brings trajectory closer to heat rate constraint). The condition of (10) is evaluated onboard and once it is satisfied (at an altitude of around 50 km), the transition is made to the guidance law. The guidance law is then tasked to guide the vehicle from transition point to the TAEM entry point.

### 2.3. Path Constraints

Although in the presented problem a QEG flight is adopted, the associated path constraints cannot be assumed to be satisfied under disturbances. Therefore, additional measure is required to ensure satisfaction of the path constraints. The associated path constraints are heating rate, normal aerodynamic load factor, and dynamic pressure. These are expressed as

(11) $$\begin{array}{c} \dot{Q}=C\sqrt{\rho }v^{3.5}\leq {\dot{Q}}_{\max},\\ n=\frac{L}{\left(mg\right)}\leq n_{\max},\\ q=0.5\rho v^{2}\leq q_{\max}, \end{array}$$

where the maximum allowable limits of heat rate (*Q*_max⁡_), normal load (*n*_max⁡_), and dynamic pressure (*q*_max⁡_) are all specified and the term *C* in (11) is a given constant.

### 2.4. Terminal Constraints

Terminal constraints are set as restrictions on the vehicle's position, angle, and velocity as per the requirements for TAEM, where terms with the superscript “*d*” denote desired values and those with subscript “*f*” represent actual terminal values:

(12) $$\begin{array}{c} r_{f}-r^{d}\approx 0,\;\theta_{f}-\theta^{d}\approx 0,\;\varphi_{f}-\varphi^{d}\approx 0,\\ \gamma_{f}-\gamma^{d}\approx 0,\;v_{f}-v^{d}\approx 0,\;\psi_{f}-\psi^{d}\approx 0. \end{array}$$

### 2.5. Physical Constraints

Besides the constraints of entry flight and TAEM requirement, the flight vehicle is assumed to have limitations on the control parameters. The vehicle needs to be guided within the following limitations:

(13) $$\begin{array}{c} \alpha_{\min}\leq \alpha_{c}\leq \alpha_{\max},\;\;\sigma_{\min}\leq \sigma_{c}\leq \sigma_{\max}. \end{array}$$

## 3. Guidance Law Design

The reason for using a Bézier approximation is to facilitate an inverse solution of the entry dynamics. The inverse problem approach is essentially the core of this method. Exposition of the method comprises the inverse dynamics formulation followed by the techniques for the Bézier approximation and the enforcement of constraints.

### 3.1. Inverse Dynamics Formulation

The first step in the inverse approach is to derive an expression for the control parameters (angle of attack *α* and bank angle *σ*) from the vehicle dynamics. In order to apply a Bézier approximation, the inverse formulation is carried out by removing dependency on time (*t*), that is, obtaining a latitude dependent system of dynamic equations, as in the following:

(14) $$\begin{array}{c} r^{\prime }=\frac{r\tan\gamma }{\cos\psi }, \end{array}$$

(15) $$\begin{array}{c} \theta^{\prime }=\tan\psi \text{sec}\varphi , \end{array}$$

(16) $$\begin{array}{c} v^{\prime }=\frac{r\left(D-mg\sin\gamma \right)}{\left(mv\cos\gamma \cos\psi \right)}, \end{array}$$

(17) $$\begin{array}{c} \gamma^{\prime }=\frac{\left(rL\cos\sigma -rmg\cos\gamma +mv^{2}\cos\gamma \right)}{\left(mv^{2}\cos\gamma \cos\psi \right)}, \end{array}$$

(18) $$\begin{array}{c} \psi^{\prime }=\frac{rL\sin\sigma }{\left(mv^{2}\cos\gamma \cos\psi \right)}+\tan\psi \tan\varphi . \end{array}$$

From (17) and (18), expressions for the acceleration commands can be found as

(19) $$\begin{array}{c} a_{y}=\left(\gamma^{\prime }\cos\psi +\frac{rg}{v^{2}}+1\right)\left(\frac{mv^{2}\cos\gamma }{r}\right), \end{array}$$

(20) $$\begin{array}{c} a_{z}=\left(\psi^{\prime }-\tan\psi \tan\varphi \right)\left(\frac{mv^{2}\cos^{2}\gamma \cos\psi }{r}\right), \end{array}$$

where

(21) $$\begin{array}{c} \gamma^{\prime }=\cos^{2}\gamma \left(r^{\prime \prime }\cos\psi -r^{\prime }\psi^{\prime }\sin\psi -\frac{r^{\prime 2}\cos\psi }{r}\right), \end{array}$$

(22) $$\begin{array}{c} \psi^{\prime }=\cos^{2}\psi \left(\theta^{\prime \prime }\cos\varphi -\theta^{\prime }\varphi^{\prime }\sin\varphi \right). \end{array}$$

Analysis of (19) and (20) indicate that the guidance command of the vehicle depends on the shape of the trajectory, through the terms *θ*′′ and *φ*′′. This deduction leads to the opinion that the guidance commands can be solved explicitly if the derivative of an approximation of the entry trajectory is available.

### 3.2. Approximation of Entry Trajectory Using the Bezier Curve

In the presented method, the entry trajectory is approximated by a Bézier curve. This is because the derivative of this approximation can be easily obtained which is shown in the formulation. Theoretically an entry trajectory can be described precisely using a Bézier curve. However, the degree of the curve depends on the entry trajectory. For some trajectories a 2nd-order or 3rd-order curve may suffice, whereas a curve with a higher degree of freedom may be required for other entry trajectories. As such, the degree of the curve depends on the trajectory profile. The method proposed in this paper is applicable only for a Bezier curve of 3rd degree. As such, for curves with higher degree of freedom, the trajectory needs to be partitioned into 3rd degree Bézier curves.

Mathematically, a Bézier curve is defined as a parametric curve *P*(*τ*) that is a polynomial function of the parameter *τ* ∈ [0,1]. The matrix form of expression for a Bézier curve is

(23) $$\begin{array}{c} P\left(\tau \right)=\left[\tau^{n},\tau^{n-1},\ldots ,\tau ,1\right]\left[N\right]{\left[P_{0},P_{1},\ldots ,P_{n-1},P_{n}\right]}^{T}, \end{array}$$

where *P*_*i*_ (*i* = 0,1,…, *n* − 1) are the control points of the curve, *n* is the number of these points, and [*N*] is a basis matrix which depends on the degree of the curve and is expressed [20] as

(24) $$\begin{array}{c} N=\left[\begin{array}{cccc} \left(\begin{array}{c} n\\ 0 \end{array}\right)\left(\begin{array}{c} n\\ n \end{array}\right){\left(-1\right)}^{n} & \left(\begin{array}{c} n\\ 1 \end{array}\right)\left(\begin{array}{c} n-1\\ n-1 \end{array}\right){\left(-1\right)}^{n-1} & \cdots & \left(\begin{array}{c} n\\ n \end{array}\right)\left(\begin{array}{c} n-n\\ n-n \end{array}\right){\left(-1\right)}^{0}\\ \left(\begin{array}{c} n\\ 0 \end{array}\right)\left(\begin{array}{c} n\\ n-1 \end{array}\right){\left(-1\right)}^{n-1} & \left(\begin{array}{c} n\\ 1 \end{array}\right)\left(\begin{array}{c} n-2\\ n-2 \end{array}\right){\left(-1\right)}^{n-2} & \cdots & 0\\ \vdots & \vdots & \cdots & 0\\ \left(\begin{array}{c} n\\ 0 \end{array}\right)\left(\begin{array}{c} n\\ 1 \end{array}\right){\left(-1\right)}^{1} & \left(\begin{array}{c} n\\ 1 \end{array}\right)\left(\begin{array}{c} n-1\\ 0 \end{array}\right){\left(-1\right)}^{0} & \cdots & 0\\ \left(\begin{array}{c} n\\ 0 \end{array}\right)\left(\begin{array}{c} n\\ 0 \end{array}\right){\left(-1\right)}^{0} & 0 & \cdots & 0 \end{array}\right]. \end{array}$$

**Figure 1:**
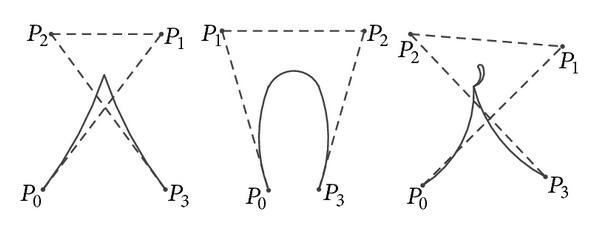
Bézier curves of third degree and their control polygons.

The degree of a Bézier curve depends on the number of points (i.e., control points) which are used to define it.  Figure 1 shows Bézier curves of third degree where the control points are *P*_0_, *P*_1_, *P*_2_, and *P*_3_. Only defining these control points complete the Bézier curve. Bézier curves can be of any number of degrees. The more the number of degrees is, the more flexible the curve is. In the method of this paper, a third-degree curve (defined by 4 control points) is selected.

In the proposed work, the entry trajectory is approximated by a Bezier curve in a local 3D coordinate system. As such, two coordinate transformations are required, first from the geodetic to the earth-centered-earth-fixed, and then to the local coordinate frame as shown in (25) [21]. Consider

(25) {xeyeze}=r{cos⁡φsinθsinφcos⁡φcos⁡θ},{xyz}={−sinθ0cos⁡θ00−cos⁡φ0cos⁡θ0−cos⁡φ0sinθ0−sinθ0−sinφ0cos⁡θ0−sinφ0sinθ0cos⁡φ0}×{xe−x0ye−y0ze−z0}.

After the above transformations, the vehicle trajectory can be represented as three Bézier curves as shown in Figure 5, each being projections of it on the planes *xy*, *yz*, and *zx*:

(26) x(τ)=[τ3,τ2,τ,1][N3][Px0,Px1,Px2,Pxf]T,y(τ)=[τ3,τ2,τ,1][N3][Py0,Py1,Py2,Pyf]T,z(τ)=[τ3,τ2,τ,1][N3][Pz0,Pz1,Pz2,Pzf]T,

where ^3^ *N* is the Bézier basis matrix of third order, and the term *τ* and the end control points *P*_*x*0_, *P*_*xf*_, *P*_*y*0_, *P*_*yf*_, *P*_*z*0_, and *P*_*zf*_ can be defined using the properties of a Bézier curve:

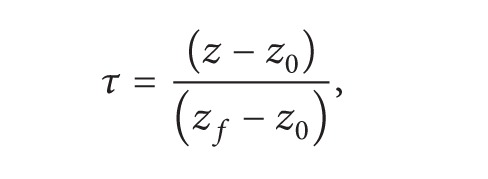
(27)

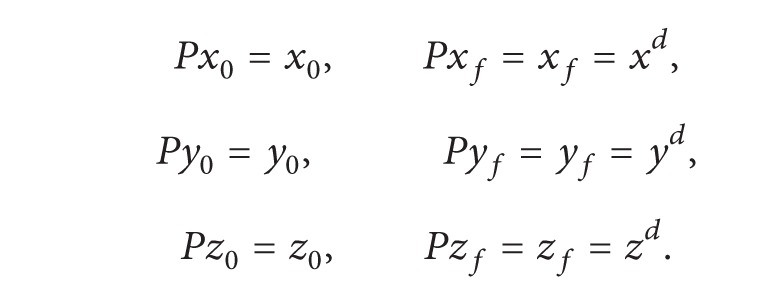
(28)


**Figure 2:**
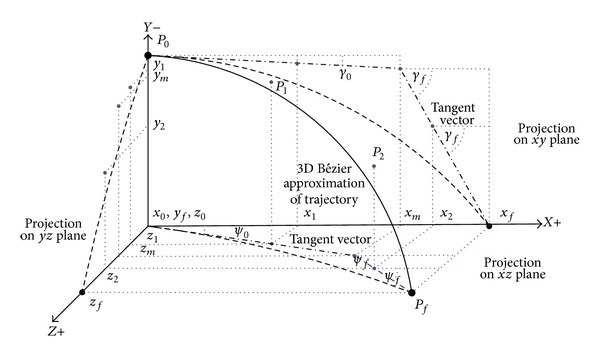
3D Bézier curve and its control points.

**Figure 3:**
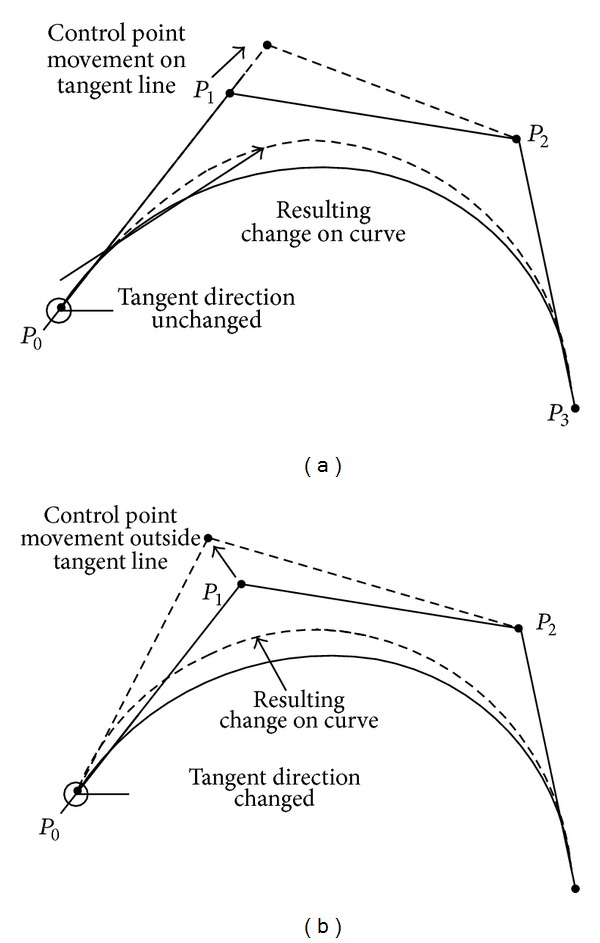
Effect of the adjustment of interior control points. Direction of the tangent of the curve at the initial control point remains unchanged if the interior control point is adjusted on the same tangent line. Moving the control point outside of the tangent line changes the tangent and its direction.

The approximated Bézier curve and its control points are shown in Figure 2 along with their projections. According to the properties of a Bézier curve, the interior control points *P*_1_ and *P*_2_ lie on the tangents of the curve at points *P*_0_ and *P*_*f*_. From the projection on the *xy* plane, it is evident that the interior control points satisfy the initial and terminal flight path angle constraints through *x* and *y* coordinates, whereas the constraints of azimuth angle are satisfied through the *z* coordinate. As such, defining the coordinates of *P*_1_ and *P*_2_ completes the approximation of a constrained trajectory. However, these points need to be defined such that these remain on the tangent lines. This, as explained in Figure 3, ensures that the boundary conditions for flight path angle and azimuth angle are met.

**Figure 4:**
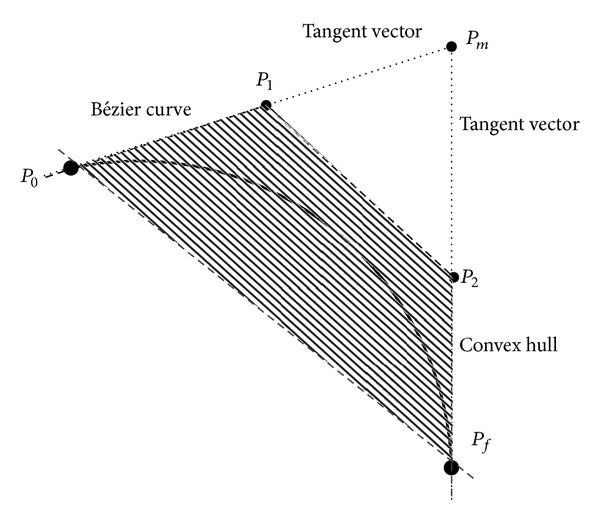
Bézier parameter representation.

The potential of adjusting a Bézier curve, while keeping the end points and directions unchanged, is very significant. This means that if a flight trajectory can be approximated as a Bézier curve, then it is possible to adjust its flight by moving the interior control points along the end tangents, thus keeping end points and directions unchanged. In order to avail this technique, parameters *k*_1_ and *k*_2_ termed as “Bézier Parameter” are introduced. These parameters are used to regulate movement of the interior control points along their line of tangency.  Figure 4 illustrates the Bézier parameters. In the figure, *P*_*m*_ is the intersection point of the tangent lines *P*_0_ *P*_*m*_ and *P*_*f*_ *P*_*m*_. Representation of Bézier parameter *k*_1_ as the ratio of (*P*_1_ − *P*_0_) and (*P*_*m*_ − *P*_0_) facilitates sliding of *P*_1_ on the tangent line *P*_*m*_ *P*_0_ with changing values of *k*_1_. Thus, if only *k*_2_ is changed, control points *P*_0_, *P*_1_, *P*_*f*_ and tangent lines *P*_0_ *P*_*m*_ and *P*_*f*_ *P*_*m*_ stay unchanged in their position and direction.

Mathematically, the Bezier parameters can be defined as

(29) $$\begin{array}{c} k_{1}=\frac{\left({P_{x}}_{1}-{P_{x}}_{0}\right)}{\left({P_{x}}_{m}-{P_{x}}_{0}\right)},\;\;k_{2}=\frac{\left({P_{x}}_{2}-{P_{x}}_{m}\right)}{\left({P_{x}}_{f}-{P_{x}}_{m}\right)}. \end{array}$$

Using the Bézier parameters, the following equations can be derived for the intersection point and interior control points:

(30) $$\begin{array}{c} P_{ym}=\frac{\tan\gamma_{0}\tan\gamma_{f}}{\tan\gamma_{f}-\tan\gamma_{0}}\left(P_{xf}-P_{x0}+\frac{P_{yf}}{\tan\gamma_{f}}-\frac{P_{y0}}{\tan\gamma_{0}}\right),\\ P_{xm}=P_{x0}+\frac{\left(P_{ym}-P_{y0}\right)}{\tan\gamma_{0}},\\ P_{x1}=k_{1}\left(P_{xm}-P_{x0}\right)+P_{x0},\\ P_{x2}=k_{2}\left(P_{xf}-P_{xm}\right)+P_{xm},\\ P_{y1}=P_{y0}+\left(P_{x1}-P_{x0}\right)\tan\gamma_{0},\\ P_{y2}=P_{yf}+\left(P_{x2}-P_{xf}\right)\tan\gamma_{f},\\ P_{z1}=P_{z0}-\left(P_{x1}-P_{x0}\right)\tan\psi_{0},\\ P_{z2}=P_{zf}+\left(P_{xf}-P_{x2}\right)\tan\psi_{f}. \end{array}$$

For a terminally constrained flight, it is thus possible to complete the approximation provided that appropriate values of the parameters *k*_1_ and *k*_2_ are known. These values can be obtained through optimization. The trajectory can then be represented by the initial and terminal states (*P*_0_, *P*_*f*_, *γ*_0_, *ψ*_0_, *γ*_*f*_, *ψ*_*f*_) and the Bézier parameters (*k*_1_, *k*_2_). With the approximated Bezier curve available, the first and second derivatives of the entry vehicle's position vectors *x*(*τ*), *y*(*τ*), and *z*(*τ*) can be found as follows:

(31) dxdτ=[3τ22τ10][N3][Px0Px1Px2Pxf]T,dydτ=[3τ22τ10][N3][Py0Py1Py2Pyf]T,dzdτ=[3τ22τ10][N3][Pz0Pz1Pz2Pzf]T,d2xdτ2=[6τ200][N3][Px0Px1Px2Pxf]T,d2ydτ2=[6τ200][N3][Py0Py1Py2Pyf]T,d2zdτ2=[6τ200][N3][Pz0Pz1Pz2Pzf]T.

Using the above equations, the previously unknown terms of (20) and (21) can then be found using the following relations:

(32) $$\begin{array}{c} r^{\prime }=\frac{\left(dy/d\tau \right)}{\left(dz/d\tau \right)},\;\;\theta^{\prime }=\frac{\left(dx/d\tau \right)}{\left(dz/d\tau \right)},\;\;\varphi^{\prime }=\frac{\left(dz/d\tau \right)}{\left(dz/d\tau \right)},\\ r^{\prime \prime }=\frac{\left\{\left(d^{2}y/d\tau^{2}\right)-\left(dy/d\tau \right)\left(d^{2}z/d\tau^{2}\right)/\left(dz/d\tau \right)\right\}}{{\left(dz/d\tau \right)}^{2}},\\ \theta^{\prime \prime }=\frac{\left\{\left(d^{2}x/d\tau^{2}\right)-\left(dx/d\tau \right)\left(d^{2}z/d\tau^{2}\right)/\left(dz/d\tau \right)\right\}}{{\left(dz/d\tau \right)}^{2}}. \end{array}$$

### 3.3. Enforcement of Constraints

In the formulation presented so far, the constraints of terminal position are addressed through (28). The constraints on flight path and azimuth angles are dealt through (30). The normal load constraint is imposed on the acceleration command as follows:

(33) ay=aynmax⁡gay2+az2,az=aznmax⁡gay2+az2,if ay2+az2g≥nmax⁡.

From the above acceleration commands, the angle of attack and bank angle commands can be found:

(34) αc=f−1(cl)=f−1(may(qSref)),σc=tan−1(azay).

Heat rate and dynamic pressure constraints (11) are transformed into an angle of attack constraint by employing QEGC. The lower limits on the angle of attack angle ensure satisfaction of the constraints:

(35) αmin⁡q=f−1(clmin⁡q)=f−1mv(g/v+ε−v/r)qmax⁡Sref,αmin⁡Q=f−1(clmin⁡Q)=f−12mC2v5(g/v+ε−v/r)Q˙max⁡2Sref.

The final control command may then be obtained as follows:

(36) $$\begin{array}{c} \alpha_{c}=\{\begin{array}{cc} \alpha_{\max} & \text{if}\;\;\alpha_{c}>\alpha_{\max}\\ \alpha_{\min} & \text{if}\;\;\alpha_{c}<\alpha_{\min}\\ \alpha_{\min}^{q} & \text{if}\;\;\alpha_{c}<\alpha_{\min}^{q}\\ \alpha_{\min}^{Q} & \text{if}\;\;\alpha_{c}<\alpha_{\min}^{Q}. \end{array}\\ \sigma_{c}=\{\begin{array}{cc} \sigma_{c} & \text{if}\;\;\sigma_{\min}\leq \sigma_{c}\leq \sigma_{\max}\\ \sigma_{\max} & \text{if}\;\;\sigma_{c}>\sigma_{\max}\\ \sigma_{\min} & \text{if}\;\;\sigma_{c}>\sigma_{\max}. \end{array} \end{array}$$

The control command thus obtained complies with all path and boundary constraints except that of velocity. For velocity constraint, a technique is proposed. In this approach, a relation between terminal velocity and the parameter *k*_2_ is found, which is then used for adjusting *k*_2_ as per the velocity requirement. The details regarding this method are explained in the following section.

## 4. Guidance Method Implementation

The guidance method is implemented in two steps. The first part is the parameter optimization process where the Bézier parameters *k*_1_ and *k*_2_ are obtained off board. The onboard implementation then follows, which includes a neural network assisted module for velocity control. The details of the complete method are explained here.

### 4.1. Parameter Optimization

**Figure 5:**
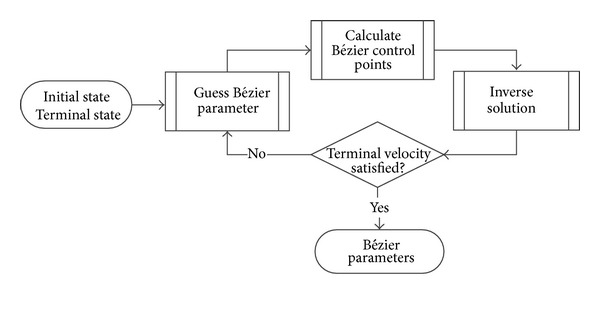
Off board parameter optimization process.

The parameter optimization process is aimed at obtaining the parameters *k*_1_ and *k*_2_ for representing a feasible trajectory. This process is similar to trajectory optimization, which is used in profile tracking guidance. The difference is that the proposed method searches for a feasible and constrained trajectory in terms *k*_1_ and *k*_2_. Additionally, the search space in the proposed technique is significantly limited. For instance, for a QEG trajectory, a search space in the range of [0.4~1] is found to be adequate. Within these ranges, guesses are made for the values of *k*_1_ and *k*_2_. Using these guess values and the boundary conditions, an inverse solution can be obtained. This solution is in turn checked against the terminal velocity constraint. Once this requirement is fulfilled, the optimization process is terminated. An illustration of the process is shown in Figure 5.

### 4.2. Adjustment of *k*_2_

**Figure 6:**
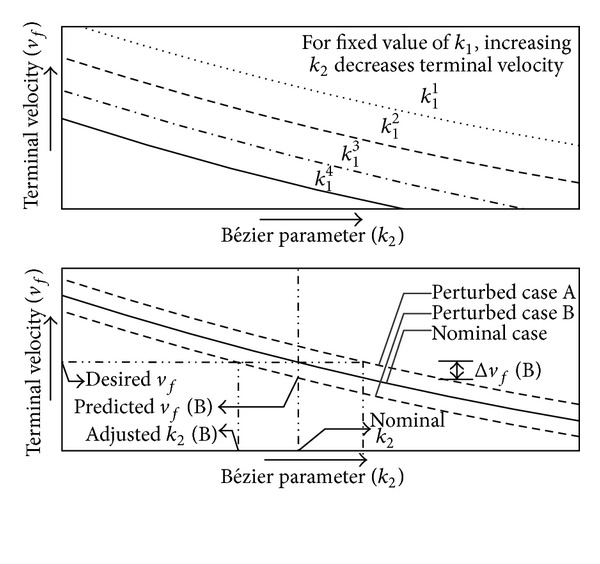
Velocity control through the adjustment of Bézier parameter *k*_2_.

Satisfaction of the terminal velocity constraint is ensured by adjusting the parameter *k*_2_. The effect of *k*_2_ on the terminal velocity is explained in Figure 6. The adjustment is made by using a polynomial expression of *k*_2_ with respect to *v*_*f*_. This polynomial expression as in (37) can be generated during the parameter optimization:

(37) $$\begin{array}{c} k_{2_{\text{nominal}}}^{\text{adjust}}=av_{f}^{2}+bv_{f}+c. \end{array}$$

The polynomial in (37) can be used for a nominal flight only. In the case of a perturbed flight, the polynomial relation is supposed to vary. Analyses of several cases indicate that the change in the resulting terminal velocity due to perturbation remains almost the same for all values of *k*_2_. Following this deduction, the relation in (38) is used for adjusting *k*_2_, where *v*_*fn*_ and *v*_*fp*_ are the terminal velocities for the nominal and perturbed cases:

(38) $$\begin{array}{c} k_{2}^{\text{adjust}}=a{\left(2v_{fn}-v_{fp}\right)}^{2}+b\left(2v_{fn}-v_{fp}\right)+c. \end{array}$$

Using the above relation, a desired terminal velocity can be maintained through *k*_2_ adjustment given that *v*_*fp*_ is known. For this, a neural network is used to predict terminal velocity in the presence of perturbations.

### 4.3. Neural Network

**Figure 7:**
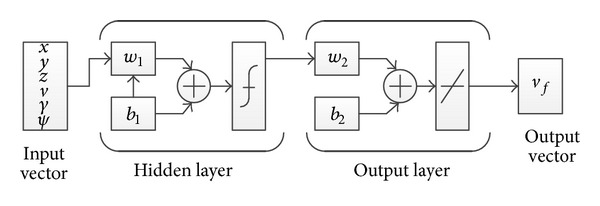
Architecture of feed forward network.

Artificial neural networks (ANN) are inspired by the biological neural systems. These networks are composed of artificial neurons which are designed to receive input and generate activation signal which in turn triggers an output. A network of these artificial neurons can be trained to solve complex problems. In several research on entry guidance methods [22, 23], application of ANN has been made. In the present work, an ANN is used for predicting terminal velocity based on the current state. For this purpose, a two-layer feed forward back propagation network is used. The ANN is designed to receive vehicle states as input and return terminal velocity as the output. Architecture of the ANN is shown in Figure 7. As in a biological brain, ANNs learn through training. In order to train the ANN, a set of training data is required. In the proposed method, the training data set consists of current state parameters and the corresponding terminal velocity outputs. For generating the data, a number of simulations with nominal values of *k*_1_ and *k*_2_ were performed for different perturbations. Based on this training, the ANN provides prediction of the terminal velocity and accordingly *k*_2_ is adjusted. Employing ANN can substantially reduce the time needed for onboard computation of terminal velocity.

### 4.4. Onboard Guidance Law Implementation

**Figure 8:**
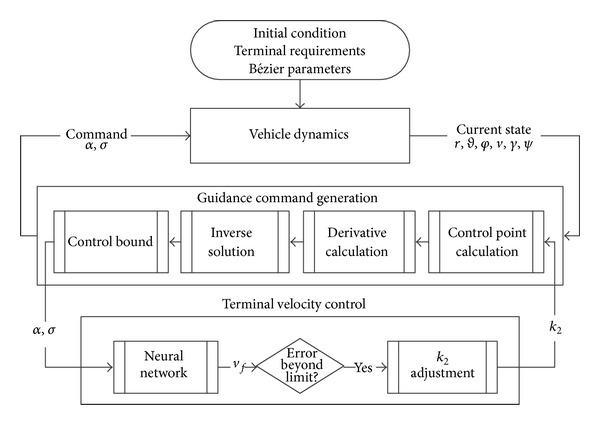
Guidance law implementation.

The onboard implementation starts with inputs of initial state, terminal state, and the optimized Bézier parameters. A flow chart of the process is shown in Figure 8. At every guidance cycle, the control points are recalculated for the current position, which are then used to solve the inverse system. In brief, the following steps are performed.

1. From the current state (*r*, *θ*, *φ*, *γ*, *ψ*), the control points *P*_*x*0_, *P*_*y*0_, *P*_*z*0_ are defined.
2. Using the stored values of *k*_1_ and *k*_2_, the interior control points are calculated.
3. The acceleration commands are obtained and the path constraints are applied.
4. At specified intervals, the terminal velocity is predicted using the ANN, and if necessary, *k*_2_ is adjusted accordingly.

## 5. Guidance Law Evaluation

Performance and robustness of the guidance law have been evaluated through simulations of full nonlinear dynamics for Lockheed-Martin's CAV-H vehicle [24]. It is a typical hypersonic high lift entry vehicle with a weight of 907 kg and a reference area of 0.4839 m^2^. The aerodynamic model of the vehicle was taken from [25]. In the evaluation, a nominal profile is selected and simulations are carried out considering atmospheric disturbances, model uncertainties (lift and drag coefficients), and initial state perturbations.

### 5.1. Nominal Profile and Perturbations

A nominal trajectory was generated for the desired boundary conditions using the off board parameter optimization process. The specified boundary conditions and the obtained Bézier parameters are shown in Table 1.

### 5.2. Neural Network Design

**Figure 9:**
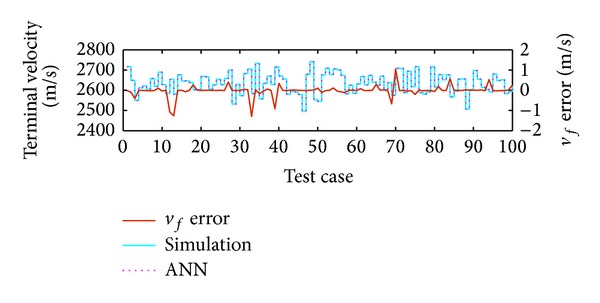
Accuracy of ANN in predicting terminal velocity.

The ANN for the prediction of the terminal velocity was set up using the neural network toolbox of MATLAB. A two-layer feed forward back propagation network was used with 1000 neurons and it was trained for 1000 cases. Comparison of the ANN result with actual simulation data indicated 99% accuracy as shown in Figure 9. It should be mentioned that accuracy of ANN prediction depends on the number of neurons.

### 5.3. Polynomial for Terminal Velocity Control

**Figure 10:**
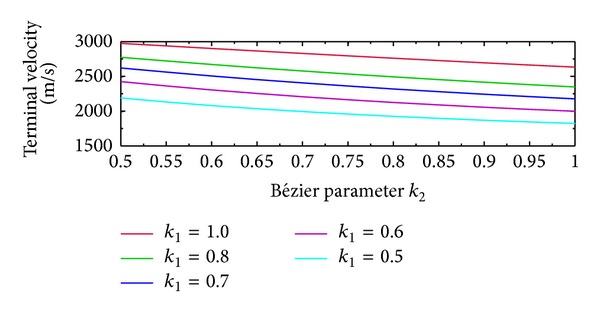
Terminal velocity and *k*_2_, *k*_1_ plot.

The polynomial expression of *k*_2_ with respect to *v*_*f*_. was generated during parameter optimization. The obtained coefficients for the polynomial were *a* = 2.32 × 10^−7^, *b* = −0.0024, and *c* = 5.46.  Figure 10 shows the effect of the Bézier parameters (*k*_1_ and *k*_2_) on the terminal velocity. The plots indicate that the increase in the value of *k*_1_ increases the range of achievable terminal velocity. Within this range, an increase in *k*_2_ reduces the terminal velocity.

### 5.4. Simulation Results

Simulations were carried out for 500 cases with normally distributed errors in the initial state and random ±10% error in aerodynamic and atmospheric modeling. The perturbation specifications are shown in Table 2. Independent random variables with normal distribution were used to model the initial perturbations. The dispersion ranges shown in Table 2 are three standard deviation (3*σ*) values.

**Figure 11:**
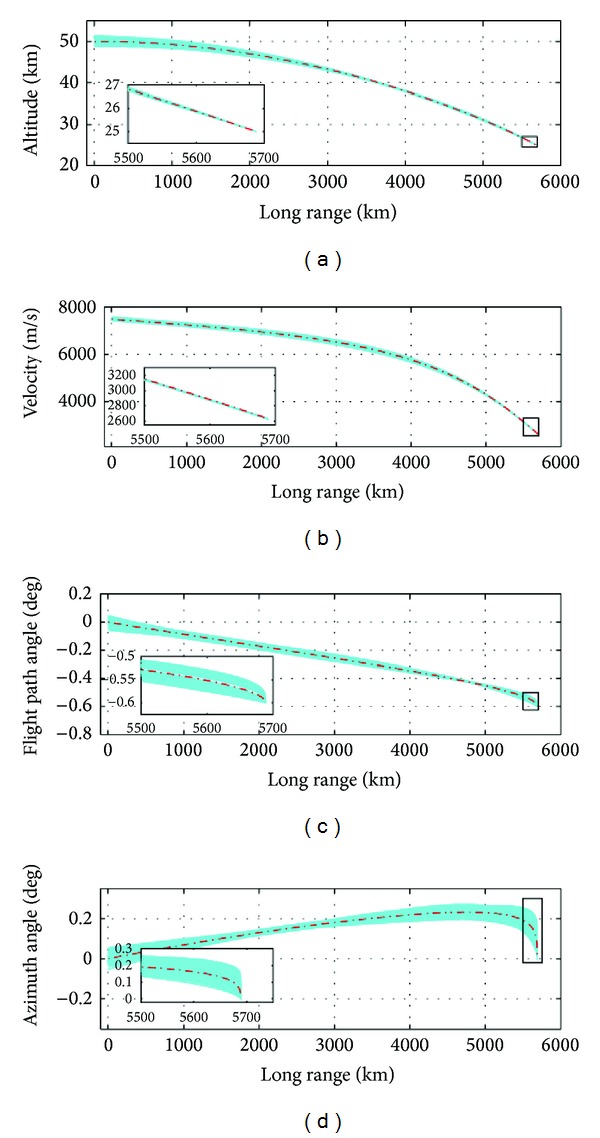
Simulation results. (a) Altitude, (b) velocity, (c) flight path angle, and (d) azimuth angle.

**Figure 12:**
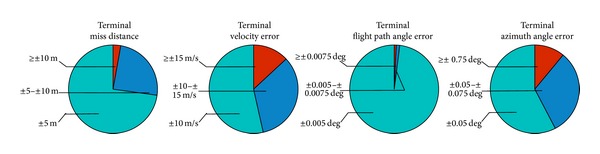
Statistics of terminal errors for the 500 runs.

The state plots from the 500 cases are shown in Figure 11. The terminal errors in the state have been plotted in Figure 12. For ease of understanding, the statistical result has been given in Table 3.

**Figure 13:**
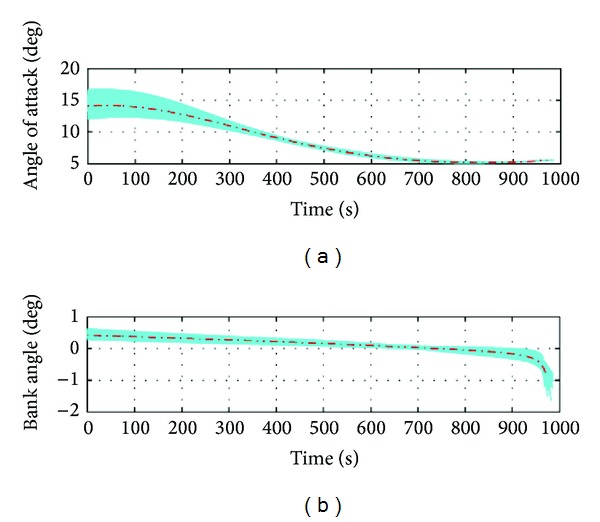
Control history from the simulations. (a) Angle of attack profile and (b) bank angle profile.

Graphical representation of the terminal error statistics is shown in Figure 13. Considering the results, the proposed method seems effective in meeting terminal constraints under perturbations and uncertainties.

**Figure 14:**
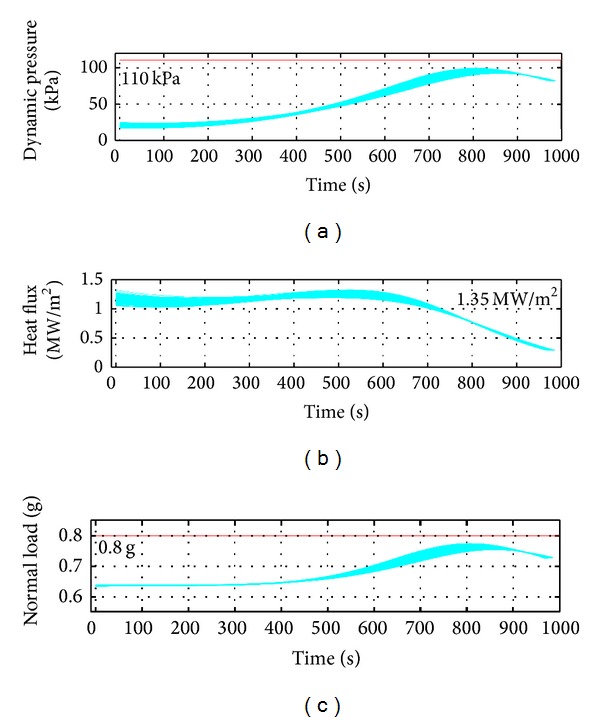
Path constraint plot for the runs. (a) Dynamic pressure remains within 110 kPa, (b) heating rate stays within 1.5 MW/m^2^, and (c) the normal load is within 1 g.

Attack and bank angle profiles corresponding to the Monte Carlo simulation are plotted in Figure 13, with an indication of the nominal control profile. The plot shows smooth control history. The plots of dynamic pressure, heat flux, and normal load are shown in Figure 14. The simulations were carried out with the maximum value of these constraints set as 200 k Pa, 2 MW/m^2^, and 2 g, respectively. The results show for all the cases that the constraints were met with adequate margin remaining.

**Figure 15:**
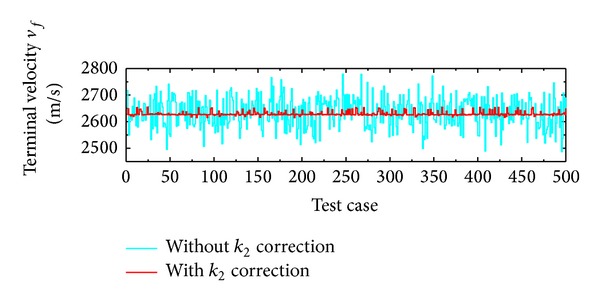
Effect of terminal velocity control through *k*_2_ adjustment.

The result shown in Figure 11 shows the terminal velocity error to be within 25 m/sec, which suggests high precision. This accuracy is possible due to the technique used for maintaining the terminal velocity constraint.  Figure 15 shows the plot of terminal velocity errors for the 500 cases run with (red colored) and without (cyan colored) adjustment of *k*_2_. The adjustment appears to have achieved a remarkable reduction in the velocity error.

## 6. Conclusions

In this paper, an inverse guidance law is presented. The central aspect of the method is the representation of a constrained trajectory using a Bézier curve. In the formulation, the approximation is made by using two proposed parameters. These parameters are derived from the Bézier control points and are utilized for ensuring boundary constraints. The method also employs ANN for predicting and controlling terminal velocity. The proposed method is evaluated through a 500-run simulation considering perturbations in the initial state, and error in aerodynamic and atmospheric modelling. The results indicate that the guidance scheme performs remarkably in satisfying terminal and path constraints. The presented method is however limited to trajectories with limited curvature variation. Further research on use of a higher degree Bézier curve and a faster parameter optimization process can increase the method's applicability in global payload delivery systems as well as future planetary missions.

## Figures and Tables

**Table 1:** Nominal profile and Bézier parameters.

Nominal profile		
Parameter	Initial position	Terminal position
Altitude	50 km	25 km
Velocity	7500 m/sec	2630 m/sec
Flight path angle	−0.001°	−0.6°
Azimuth angle	0°	0°

Parameters	*k*_1_	*k*_2_

Bézier parameters	0.848	0.717

**Table 2:** The perturbations in Monte Carlo run.

Parameter	Value
Range	±1.5 km
Altitude	±1.5 km
Cross range	±1.5 km
Aerodynamic modelling	±10%
Velocity	±75 m/sec
Flight path angle	±0.06°
Azimuth angle	±0.06°
Atmospheric modelling	±10%

**Table 3:** Monte Carlo simulation results.

Parameter	Miss distance	Δ*γ*_*f*_	Δ*ψ*_*f*_	Δ*v*_*f*_
Maximum	159 m	0.002°	0.02°	19.8 m/sec
Mean	0.22 m	0.0001°	0.002°	5.01 m/sec
Std. deviation	4.54 m	0.001°	0.003°	7.23 m/sec
